# The role of bariatric surgery in liver transplantation: timing and type

**DOI:** 10.1007/s00423-022-02606-5

**Published:** 2022-07-19

**Authors:** Danial Safavi, Ben Creavin, Tom K. Gallagher, Michael E. Kelly

**Affiliations:** 1 Royal College of Surgeon, Dublin, Ireland; 2 Department of Surgery, University Hospital Waterford, Waterford, Ireland; 3 Department of Hepatobiliary Surgery, St Vincent’s University Hospital Dublin, Dublin, Ireland; 4 Department of Surgery, St James Hospital, Dublin, Ireland

**Keywords:** Liver transplantation, Bariatric surgery, Weight loss surgery, Sleeve gastrectomy, Gastric bypass

## Abstract

**Introduction:**

The rise in obesity worldwide has shifted the indications for liver transplantation (LT), with non-alcoholic steatohepatitis (NASH) being the second most common indication for transplantation. There remains an underestimation of cirrhosis being attributed to NASH. Bariatric surgery (BS) is a reliable solution to overcome obesity and its associated comorbidities. The role of BS in LT has been investigated by different studies; however, the type of BS and timing of LT need further investigation.

**Methods:**

A systemic review examining the role of BS in LT patients was performed. After selection of the studies based on inclusion and exclusion criteria, data extraction was performed by two independent reviewers. Primary outcomes included patient and graft survival.

**Results:**

From a total of 2374 articles, five met the prefined criteria. One hundred sixty-two patients had both BS + LT and 1426 underwent LT alone. The percentage of female patients in the BS + LT and LT cohorts was 75% and 35% respectively. The average age in BS + LT and LT cohorts was 43.05 vs. 56.22 years respectively. Patients undergoing BS had comparable outcomes in terms of overall patient survival, graft survival and post-operative morbidity compared to LT alone. When comparing BMI change in patients with prior versus simultaneous BS + LT, no significant difference was found.

**Conclusion:**

BS and LT patients achieve comparable outcomes to general LT populations. Further studies examining simultaneous BS + LT are needed to answer questions concerning patient selection and timing of surgery.

**Supplementary Information:**

The online version contains supplementary material available at 10.1007/s00423-022-02606-5.

## Introduction

The prevalence of obesity is rapidly rising across the world, with more than one-third of Western populations now being classified as obese [1]. The complications from the obesity epidemic include increased rates of cardiac disease, diabetes mellitus, hypercholesterolemia-related problems and metabolic disorders [2]. Over 80% of obese patients suffer from non-alcoholic fatty liver disease (NAFLD). Of these, one-third will progress to non-alcoholic steatohepatitis (NASH), and one-quarter of NASH patients develop cirrhosis [3, 4]. NASH-related cirrhosis is predicted to become the leading indication of liver transplantation over the next decade [5].

Obesity can complicate liver transplantation (LT) by increasing morbidity due to higher infection rates and wound healing issues. But increasingly, concerns over NASH development in the transplanted organ due to immunosuppressive medications and/or elevated body mass index (BMI) have been raised [6, 7]. Historically, a BMI > 40 was considered a relative contraindication in many centres [8, 9], but with the rise in adiposity, this has been reconsidered. Lifestyle modification and/or medications still remain first-line in the management of obesity, but the role of bariatric surgery is important due to its longer-lasting effect and efficacy [10]. Recent studies have shown that bariatric surgery can stop the progression, or even reverse NAFLD [11]. Of the various techniques available, sleeve gastrectomy (SG) and Roux-en-Y bypass (RYBP) remain the most commonly performed. SG is solely a restrictive procedure, whereas RYBP is both a malabsorptive and a restrictive procedure [12]. Both have observed comparable weight loss and impact on comorbidities at mid- to long-term follow-up [13].

Recently, the role of bariatric surgery before or concomitant with LT has been explored. However, there remains no consensus on the timing of BS. Theoretically, performing BS prior to LT means that the patient may reduce weight (and improve associated comorbidities); however, it requires two separate operations and hospitalisations. Alternatively, concomitant BS and LT have the benefit of one anaesthetic, with shorter hospital stay, and prevent delays to LT [14]. This review aims to address the benefit of BS prior to LT, focusing on the benefits specific to the timing and type of BS.

## Methods

A systematic review was performed according to the preferred reporting items for systematic reviews and meta-analyses checklist (PRISMA). No approval was required from the review board.

### Search strategy

An electronic search for relevant publications was performed in the PubMed database using the following search terms: “Bariatric Surgery” “Liver transplantation”, “Sleeve gastrectomy” “Liver transplant”, “Gastric bypass” “Liver transplant”, “Weight loss surgery” “Liver transplant” and “Bariatric surgery, Liver surgery”. A total of 2374 articles were yielded, all titles were initially screened and appropriate abstracts were reviewed. The last date of the search was February 30, 2021.

This systematic review was based on the following PICO (Population, Intervention, Comparison, Outcomes) criteria:

- Population: obese patients who are candidates for LT
- Intervention: bariatric surgery before or simultaneous with LT
- Comparators: LT without bariatric surgery
- Outcomes: overall survival, graft survival, post-operative morbidities after LT, length of hospital stay (LOS) after LT, change in BMI, recurrence of steatosis

### Inclusion criteria

To be included in the analysis, the studies had to meet the following criteria: (a) report on patients with both liver transplantation and bariatric surgery; (b) had a concurrent cohort that underwent only LT; (c) report on surgical and outcome measures mentioned below (patient survival, graft survival, length of stay, complications).

### Exclusion criteria

Studies were excluded from the analysis if (a) they were systematic reviews or meta-analysis; (b) they did not contain both bariatric surgery and liver transplantation; (c) outcomes of interest were not reported; (d) they involved less than 10 patients in the study.

### Data extraction

Two reviewers (DS, BC) independently reviewed the literature based on the above pre-defined strategy and criteria. Each reviewer extracted the following data variables: title and reference details (first author, journal, year and the study timeline), study population characteristics (number in the study, BMI at LT, model for end-stage liver disease (MELD) score, indication for LT), treatment specifics (type of bariatric surgery, timing of the BS relative to LT). All data were recorded independently by the literature reviewers in separate databases and were compared at the end of the reviewing process to limit selection bias. Duplicates were removed and any disparities were clarified. All median values were converted to mean and standard deviation (SD) using Hozo’s equation [15] for meta-analysis.

### Outcomes of interest

The following outcomes were used in the review to compare the differences in the timing of bariatric surgery on liver transplantation:

Primary outcomes:

- Overall survival
- Graft survival

Secondary outcomes:

- Post-operative morbidities after LT
- Length of hospital stay (LOS) after LT
- Change in BMI
- Recurrence of steatosis

Post-operative morbidities were defined as any surgical complications occurring within 30 days post-operative, including biliary stricture, bile leakage, haemorrhage, vascular complications, infection, rejection and renal failure requiring haemodialysis.

### Statistical analysis

Statistical analysis was performed using Review Manager (RevMan) [Computer program] Version 5.3. Copenhagen: The Nordic Cochrane Centre, The Cochrane Collaboration, 2014. A meta-analysis of time-to-effect measures from each of the eligible studies, specifically hazard ratios (HRs) and overall survival (OS), was performed according to the two-stage method outlined by Tierney et al. [16]. Estimates of log hazard ratios and standard errors were obtained from the results of Cox proportional hazards regression models in each study. Study results were then combined in RevMan using the generic inverse-variance method for a pooled meta-analysis. Heterogeneity was assessed by *I*-squared statistics (*I*^2^), with > 50% being considered as considerable heterogeneity. Random-effects models were used where necessary to account for potential inter-study heterogeneity. Sensitivity analyses were carried out where appropriate. *P*-values < 0.050 were considered significant.

### Risk of bias assessment

The quality of each of the studies included in this systematic review and meta-analysis was assessed using the risk of bias in the non-randomised studies of interventions (ROBINS-I) assessment tool [17], and evaluated across the ascribed seven domains:

- Confounding
- Selection of participants into the study
- Classification of interventions
- Deviations from intended interventions
- Missing data
- Measurements of outcomes
- Selection of the reported result

The quality score rating was determined for each publication by two authors (D. S., M. K.) and the overall risk of bias measured (Table 1) (Supplementary material 1).

**Table 1:** Studies included for analysis

First author	Journal/book	Publication	Timeline	BS + LT patients	LT patients	ROBINS-I bias score
Zamora-Valdes D	*Hepatology*	2018	2006–2013	29	45	Serious
Idriss R	*Liver Transpl*	2019	1985–2017	78	156	Serious
Serrano OK	*Transplantation*	2020	1987–2017	33	99	Critical
Lefere S	*Obes Surg*	2020	2008–2018	11	177	Serious
Safwan M	*Liver Transpl*	2017	2006–2015	11	949	Critical

## Results

### *Eligible studies*

**Fig. 1:**
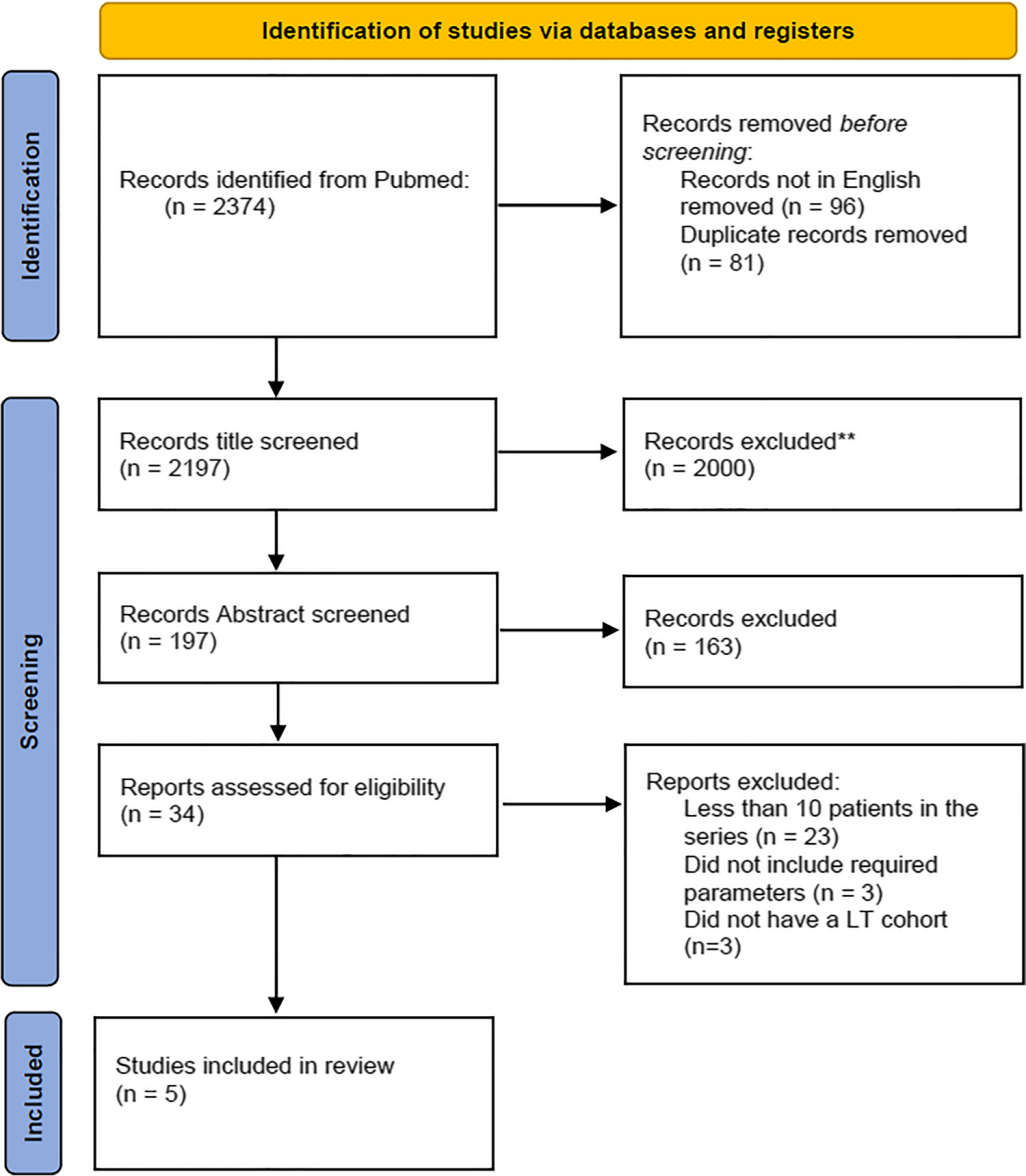
Prisma flow diagram outlining the process of study selection

A total of 2374 articles were yielded after the initial exploration of the described search phrases (Fig. 1). Initially, duplicate studies and studies not in the English language were excluded resulting in 2197 studies. After title screening, studies related to liver transplantation and bariatric surgery were selected yielding 197 studies. During abstract revision based on inclusion and exclusion criteria, 34 studies were selected for further evaluation. Based on full article revision and exclusion of studies related to other transplantations than liver, or involving less than 10 patients in their series, five articles were included for the final meta-analysis (Table 1).

All studies included two cohorts: bariatric surgery and liver transplantation (BS + LT) and liver transplantation alone (LT). There was 100% agreement between the reviewers on the data extracted.

### Patient demographics

Overall 162 patients underwent both BS and LT and 1426 had a LT alone. With regards to the timing of bariatric surgery to liver transplantation, four studies looked at patients with BS prior to LT (*n* = 133) and one study looked at BS and LT being done simultaneously (*n* = 26) [18]; none of the studies investigated BS after LT. The majority of the patients in the BS + LT cohorts were females (*n* = 122) (75%) compared to the LT-only cohort (35%); however, the number of females was not specified in two of the LT-only cohorts. The average age in the BS + LT cohort was lower than the LT cohort (43.05 years vs. 56.22 years respectively). There was a lack of uniformity in the registered data in different studies, as each recorded different parameters with different protocols. All studies registered the MELD score at transplantation, except for the LT cohort in one study. The average MELD score for the BS + LT cohorts was 19.26 and for the LT cohort 18.38.

### Types of surgery

The study performing simultaneous BS and LT utilised SG as their sole BS procedure, while others performed a variety of different approaches. Overall, the most commonly performed operations were RYBP (*n* = 82) and SG (*n* = 32), with a fewer number of patients having undergone jejunoileal bypass (JIB) (*n* = 7), biliopancreatic diversion with duodenal switch (BPD) (*n* = 4), adjustable gastric banding (AGB) (*n* = 5) and vertical banding gastroplasty (VBG) (*n* = 1) (Table 2). One of the patients with AGB and the only patient with VGB were later converted to RYBP due to inadequate weight loss before LT. One study only specified the number of patients that underwent RYBP, which may underestimate the number of patients undergoing other operations [19].

**Table 2:** Baseline of the patients undergoing: (A) BS and LT, (B) LT alone. Values expressed as absolute numbers, percentages (%) and mean (SD)

(A) Baseline of the patients undergoing BS and LT																
First author	Number of patients	Age	Female	Timing of BS to LT	Types of surgery					MELD at LT	Indication for LT					
					SG	RYBP	JIB	GB	DS		Viral hepatitis	HCC	NAFLD NASH	ALD	Autoimmune	Other
Zamora-Valdes D (2018)	29	50.7 (2.72)	58.6%	Synchronus	100%	0%	0%	0%	0%	32.0 (3.082)	3.4%	13.8%	76.9%	3.4%	6.8%	10%
Idriss R (2019)	78	53.75 (4.32)	83.3%	Before	NS	63%	NS	NS	NS	22.87 (5.34)	24.4%	NS	47.4%	19.2%	NS	1.28%
Serrano OK (2020)	33	53.42 (4.34)	78.8%	Before	6%	61%	18%	9%	3%	24 (5.2)	24%	9%	21%	27%	NS	19%
Lefere S (2020)	11	45.25 (3.18)	72.7%	Before	0%	36%	0%	27%	36%	21.1 (5.24)	0%	9%	0%	100%	0%	0%
Safwan M (2017)	11	51.3 (3)	54.5%	Before	9%	81%	9%	0%	0%	28.4 (2.588)	0%	0%	90.9%	0%	0%	0%
(B) Baseline of the patients undergoing LT only																
First author	Number of patients	Age	Female	Timing of BS to LT	Types of surgery					MELD at LT	Indication for LT					
					SG	RYBP	JIB	GB	DS		Viral hepatitis	HCC	NAFLD NASH	ALD	Autoimmune	Other
Zamora-Valdes D (2018)	45	55.4 (2.793)	55.6%	Not applicable						18.9 (2.846)	NS	NS	44.4%	NS	NS	NS
Idriss R (2019)	156	54.25 (4.33)	44.2%	Not applicable						18.25 (3.18)	31.4%	NS	47.4%	17.9%	NS	NS
Serrano OK (2020)	99	53.5 (3.18)	NS	Not applicable						25.55 (4.67)	63.6%	6%	NS	NS	NS	NS
Lefere S (2020)	177	59.7 (2.58)	22%	Not applicable						14.37 (3.55)	NS	51.10%	NS	NS	NS	NS
Safwan M (2017)	949	NS	NS	Not applicable						NS	NS	NS	NS	NS	NS	NS

### Indications for liver transplantation

The most common indications for LT in the BS + LT cohorts were NAFLD/NASH (*n* = 76), viral hepatitis (*n* = 39) and alcoholic liver disease (*n* = 36) (Table 2). In the LT cohorts, the most common indications were viral hepatitis (*n* = 112), hepatocellular carcinoma (*n* = 97) and NAFLD/NASH (*n* = 94) (Table 2). One study did not list the indication for the LT cohort.

### Surgical outcomes

**Fig. 2:**
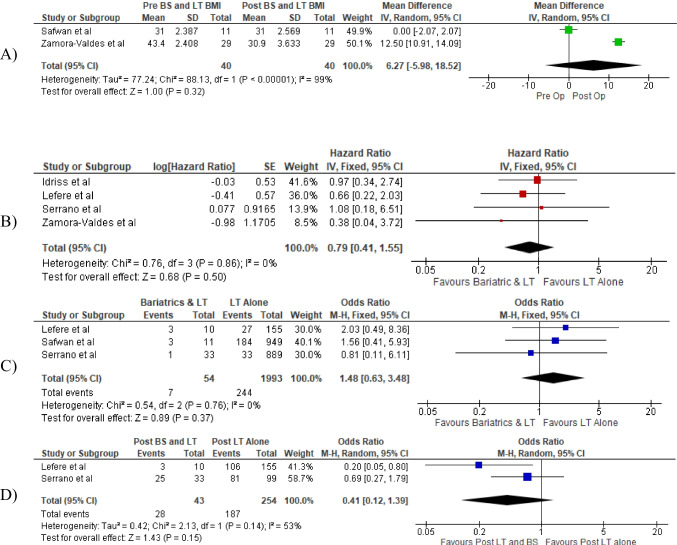
Forest plot comparison of outcomes: **A** change in BMI in patients with prior BS vs. simultaneous BS, **B** overall survival, **C** graft failure, **D** post-operative morbidity

Patient outcomes are summarised below (Table 3). Four studies did a follow-up at 3 years and one study had a follow-up at 2 years post-surgery. BMI measurements were made at the time of liver transplantation in three studies, and two of them repeated the measurement on follow-up. When comparing the BMI change between the group with prior BS and simultaneous BS + LT, no significant difference was observed (0% vs. 29%) [mean difference (MD) 6.27, 95% confidence interval (CI) − 5.98–18.52, *p* = 0.32] (Fig. 2A).

**Table 3:** Patients’ outcomes undergoing (A) BS + LT, (B) LT only. Values expressed as absolute numbers, percentages (%) and mean (SD)

(A) Outcomes of the BS + LT cohort														
First author	Number of patients	BMI at LT listing	BMI at transplant		BMI last follow-up		Hepatosteatosis		Survival		Follow-up	Graft survival	Morbidity LT	LOS after LT
Zamora-Valdes D (2018)	29	47.8 (2.121)	43.4 (2.408)		30.9 (3.633)		23.10%		93.1%		3 years	NS	31%	NS
Idriss R (2019)	78	29 (1.80)	NS		NS		NS		84%		3 years	NS	NS	14.2 (3.76)
Serrano OK (2020)	33	NS	31.27 (2.43)		NS		NS		76.40%		3 years	97%	76%	17.8 (NS)
Lefere S (2020)	11	24.825 (1.94)	NS		NS		NS		80%		3 years	70%	30%	NS
Safwan M (2017)	11	NS	31 (2.387)		31 (2.569)		18.2%		81.8%		2 years	72.7%	72.7%	10.9 (2.408)
(B) Outcomes of the LT-only cohort														
First author	Number of patients	BMI at LT listing		BMI at transplant		BMI last follow-up		Hepatosteatosis		Survival	Follow-up	Graft survival	Morbidity LT	LOS at LT
Zamora-Valdes D (2018)	45	40 (1.703)		32.7 (1.924)		38.5 (2.55)		66.70%		88.1%	3 years	NS	NS	NS
Idriss R (2019)	156	29.7 (2.82)		NS		NS		NS		82%	3 years	NS	NS	10.25 (3.19)
Serrano OK (2020)	99	NS		30.47 (2.49)		NS		NS		75.9%	3 years	96.3%	81.8%	16.8 (NS)
Lefere S (2020)	177	27.02 (30.4)		NS		NS		NS		82.2%	3 years	82.6%	68.3%	NS
Safwan M (2017)	949	NS		NS		NS		NS		88.5%	2 years	80.6%	NS	NS

All studies reported the overall patient survival. The patient survival in BS + LT cohorts was comparable to LT-only cohorts [HR 0.79 95% CI 0.41–1.55, *p* = 0.5] (Fig. 2B). From the studies with prior BS, all but one (three studies) reported graft survival, and the difference compared to LT cohorts was not significant [odds ratio (OR) 1.48, 95% CI 0.63–3.48, *p* = 0.37] (Fig. 2C).

Morbidity after LT was listed in four studies, and included biliary stricture, bile leak, haemorrhage, vascular complications, infection, rejection and renal failure requiring haemodialysis. Only two of the four studies made a measurement also for the LT cohort, both of which had prior BS. The morbidity rates were comparable in BS + LT and LT-only cohorts [OR 0.41, 95% CI 0.12–1.39, *p* = 0.15] (Fig. 2D).

Three studies reported on LOS after LT, one of which however only registered it for the BS + LT cohort. Only the study with simultaneous BS + LT reported NASH recurrence, in which the BS + LT cohort showed lower recurrence of NASH in the graft when compared to the LT-only cohort.

### Risk of bias assessment

In the “confounding” domain, and “selection of participants” domain, the risk of bias was serious in all studies (100%). In the “classification of interventions” domain, the risk of bias was low in 2 studies (40%) and moderate for 3 studies (60%). In the “deviations from intended interventions” and “bias due to missing data” domains, the risk of bias was low in 3 studies (60%) and moderate in 2 studies (40%). In the “bias in outcome measurements” domain, the risk of bias was low in 2 studies (40%) and moderate in 3 studies (60%). In the “selection of the reported results” domain, the risk of bias was low in 2 studies (40%), moderate in 2 studies (40%) and serious in 1 study (20%). The overall risk of bias according to the ROBINS-I tool was serious in 3 studies (60%) and critical in 2 studies (40%) (Supplementary material 1).

## Discussion

Obesity has shifted the indications for LT, with NASH now on the verge of becoming the leading indication [5]. The prevalence of cirrhosis related to NASH is likely to be underestimated, as more and more cryptogenic cirrhoses are now attributed to NASH [20, 21]. Obesity is also associated with higher morbidity and mortality in major abdominal surgery, including liver transplantation [22, 23].

Solid-organ transplantation is an inherently complex procedure, which is further complicated by obesity. Many factors increase the likelihood of weight gain after solid-organ transplantation, including the long-term use of immunosuppressive medications that are required for graft survival [24]. These factors however are inevitable in the LT setting, but efforts should be placed on effective weight loss in obese patients. Weight gain after LT was shown to be strongly influenced by pre-transplant obesity [25], which in turn may lead to de novo NASH or NASH recurrence [6]. In one study, patients having LT with an indication of NASH showed a NASH recurrence of 24.9% within 5 years [26]. Another study showed a higher recurrence rate of almost 100% within 5 years, in patients that showed evidence of NASH on their pre-transplantation biopsy [27]. This has led to increased efforts to facilitate weight loss to prevent these complications.

Medical and surgical options are both available to enhance weight loss. One trial noted a 5% body weight loss in 47% of patients treated with an anti-obesity medication as opposed to 25% in the placebo arm [28]. To date, there is a lack of studies to support weight loss surgery in patients awaiting LT. Bariatric surgery is known to give faster and longer-lasting outcomes compared to medical therapies [10], and has been shown to decrease the metabolic complications in obese patients, even reversing the NAFLD [29, 30].

Data collection and interpretation in these patients pose extra challenges. Ascites can obscure BMI measurements, with patients having larger volumes of ascites creating an overestimation of their BMI [7]. Another factor to consider is whether BMI accurately reflects their nutritional status. One of the studies analysed in this article used skeletal muscle index (SMI) as a surrogate for the nutritional status of patients [19]. They found the patients that underwent BS tended to have a lower SMI and have a higher mortality while waiting for transplantation. However, SMI measurements are not routinely performed, and doing repeated follow-ups on patients can be resource-demanding, while BMI measurements can be routinely performed.

Furthermore, after BS patients achieve the same BMI as their concurrent cohorts, they still may be suffering from metabolic comorbidities. There is evidence suggesting a high BMI in itself does not contribute to worse outcomes, but the comorbidities associated with obesity do [31]. Therefore it is important to consider these confounding factors when interpreting surgical outcomes, or when matching controls for the study.

The choice of the bariatric procedure has been debated. SG has been shown to improve liver function significantly more than RYBP surgery [32]. Generally, RYBP is associated with a higher rate of morbidity and mortality compared to SG, and in the LT setting, this is no exception. In two of the selected studies, most of the complications and mortality on the waiting list were in the RYBP subgroup [19, 33]. A meta-analysis comparing SG and RYBP showed, despite the higher excess weight loss in the RYBP cohort, the resolution of comorbidities was comparable in both groups, with SG being safer than RYBP [34]. A prospective study of 63 patients investigated the role of laparoscopic SG on NAFLD, with 100% of the patients showing reversal or reduction in the stage of NAFLD, with one patient showing complete regression of cirrhosis [35]. One theory that could explain the inferior outcomes of patients undergoing RYBP is the change in the way alcohol is absorbed after a RYBP surgery, with the difference in the pharmacokinetics of alcohol absorption deteriorating the liver function. Studies have shown that RYBP is associated with a higher alcohol use disorder compared to restrictive procedures [36–38].

There is still no consensus on the timing of BS relative to LT. Mosko et al. [39] found an increase in mortality post-BS in compensated and non-compensated cirrhotic patients (OR 2.17 and 21.2 respectively). But it should be noted that in this study more malabsorptive BS were performed than restrictive BS, and as mentioned earlier, RYBP is associated with higher morbidity and/or mortality while awaiting LT [19, 33, 34]. A recent meta-analysis investigating the effect of BS in cirrhotic patients also found a higher post-operative bleeding, overall complication rate and an increased LOS in non-compensated cirrhotic patients, compared to their compensated counterparts. However, long-term mortality did not differ significantly between the two groups [40]. In a case series of 32 patients who underwent SG before LT, nine patients were either not enlisted for transplantation or delisted later as the MELD score improved so significantly that LT was deemed unnecessary [41]. This naturally has a high impact on the patients’ quality of life to avoid LT and its associated complications. Not only that, but it also reduces the number of patients waiting on the transplantation waiting list. Simultaneous BS and LT can be achieved to decrease the patient’s discomfort, and remove the need for two separate hospitalization and associated costs [14]. Still, many obstacles may impede a simultaneous procedure. Most LTs are done on an urgent basis, and many institutions may not have a bariatric surgeon available. Also, the complexity of the operation may not be suitable for all patients and strict criteria should be placed in patient selection. There are no described criteria to assess suitability for undergoing simultaneous BS and LT, and a BMI > 40 might be prohibitive depending on hospital guidelines. Many bariatric surgeons may prefer LT as a bridge to BS, to avoid complications such as bleeding and risk of decompensation and to ameliorate portal hypertension before BS. In our search strategy, none of the studies looked into patients with BS performed after LT. One of the main challenges in most centres is candidacy, as BMI > 40 is a contraindication for LT [8, 9]. BS after the LT is a challenging task; as it can be technically difficult due to adhesions, the patient will be under immunosuppression, and the operation itself may alter the absorption of immunosuppressive medication and place the graft at risk [14]. However, one study performing laparoscopic SG after LT showed that SG does not seem to affect the medication dosage after BS [42].

The lack of published reports comparing different strategies restricts the amount of data available for analysis. We acknowledge that the present review has some limitations including heterogeneity in study protocols along with lack of uniformity making analysis of the data difficult. Secondly, as highlighted earlier, patients in the BS + LT cohorts tended to be younger females, and MELD scores were not significantly high, which inevitably introduces selection bias that these outcomes may not be applicable to the general population. Analysis of data from a registry can help reduce the selection bias. In our study, the LT cohort had a lower average MELD score compared to the BS + LT cohort, which can partly be attributed to the higher number of hepatocellular carcinoma (HCC) in the LT cohort, which led to MELD exceptions for undergoing transplantation. Third, most studies included were retrospective studies, which would raise the possibility of bias or unknown cofounding variables and it is impossible to know how many operations were planned, but cancelled. Lastly, as the BMI > 40 in most centres is a prohibitive factor in performing LT, hence not enough data is available on simultaneous BS and LT to make a significant comparison possible.

In conclusion, patients undergoing BS have comparable outcomes in terms of overall patient survival, graft survival and post-operative morbidity, to patients undergoing LT alone. The comparable outcomes should however be seen in light of the fact that patients requiring BS start with a worse baseline, suffer from more comorbidities and in many centres do not qualify for LT, hence having comparable results to the general population undergoing LT is a success. When comparing BMI changes in patients with prior versus simultaneous BS + LT, no significant difference was found. Perhaps, more studies in the future can increase the data available and delineate any existing differences.

## Supplementary Information

Below is the link to the electronic supplementary material.

Supplementary file1 (DOCX 16 KB)

## Data Availability

All data generated or analysed during this study are included in the manuscript and reference list.
