# Supplementary material for: The role of bariatric surgery in liver transplantation: timing and type
Source: Langenbecks Arch Surg. 2022 Jul 19;407(8):3249–58. doi: 10.1007/s00423-022-02606-5 (PMC9722877; doi:10.1007/s00423-022-02606-5)
Supplement: Supplementary file 1 — Supplementary file1 (DOCX 16 KB) [file 423_2022_2606_MOESM1_ESM.docx]

# Supplementary material 1

Risk Bias assessment using ROBINS-I tool

Supplementary table 1- Risk Bias assessment using ROBINS-I tool

| **First Author** | **Domain 1** | **Domain 2** | **Domain 3** | **Domain 4** | **Domain 5** | **Domain 6** | **Domain 7** | **Overall** |
| --- | --- | --- | --- | --- | --- | --- | --- | --- |
| Zamora-Valdes D (2018) | Serious | Serious | Low | Low | Low | Low | Low | Serious |
| Idriss R (2019) | Serious | Serious | Low | Low | Moderate | Low | Low | Serious |
| Serrano OK (2020) | Serious | Serious | Moderate | Moderate | Low | Moderate | Serious | Critical |
| Lefere S (2020) | Serious | Serious | Moderate | Moderate | Low | Moderate | Moderate | Serious |
| Safwan M (2017) | Serious | Serious | Moderate | Low | Moderate | Moderate | Moderate | Critical |
